# Correction: Racial and geographic variation in effects of maternal education and neighborhood-level measures of socioeconomic status on gestational age at birth: Findings from the ECHO cohorts

**DOI:** 10.1371/journal.pone.0268423

**Published:** 2022-05-06

**Authors:** Anne L. Dunlop, Alicynne Glazier Essalmi, Lyndsay Avalos, Carrie Breton, Carlos A. Camargo, Whitney J. Cowell, Dana Dabelea, Stephen R. Dager, Cristiane Duarte, Amy Elliott, Raina Fichorova, James Gern, Monique M. Hedderson, Elizabeth Hom Thepaksorn, Kathi Huddleston, Margaret R. Karagas, Ken Kleinman, Leslie Leve, Ximin Li, Yijun Li, Augusto Litonjua, Yunin Ludena-Rodriguez, Juliette C. Madan, Julio Mateus Nino, Cynthia McEvoy, Thomas G. O’Connor, Amy M. Padula, Nigel Paneth, Frederica Perera, Sheela Sathyanarayana, Rebecca J. Schmidt, Robert T. Schultz, Jessica Snowden, Joseph B. Stanford, Leonardo Trasande, Heather E. Volk, William Wheaton, Rosalind J. Wright, Monica McGrath, on behalf of program collaborators for Environmental Influences on Child Health Outcomes

The third author’s name is spelled incorrectly in the manuscript and supporting information table S1 Funding. The correct name is: Lyndsay Avalos. The correct citation is: Dunlop AL, Essalmi AG, Avalos L, Breton C, Camargo CA, et al. (2021) Racial and geographic variation in effects of maternal education and neighborhood-level measures of socioeconomic status on gestational age at birth: Findings from the ECHO cohorts. PLOS ONE 16(1): e0245064. https://doi.org/10.1371/journal.pone.0245064.

The authors have provided the corrected supporting information table. Please view the correct S1 Funding below.

## Supporting information

S1 Funding Grant numbers for ECHO Awardees and Cohorts that contributed aggregate data for this analysis. (DOCX)
