# Supplementary material for: Racial and geographic variation in effects of maternal education and neighborhood-level measures of socioeconomic status on gestational age at birth: Findings from the ECHO cohorts
Source: PLoS One. 2021 Jan 8;16(1):e0245064. doi: 10.1371/journal.pone.0245064 (PMC7794036; doi:10.1371/journal.pone.0245064)
Supplement: S1 Table — (DOCX) [file pone.0245064.s001.docx]

| **Data dictionary: Single record per index delivery of child. Please leave the cell blank if 1) missing value, 2) if you have no information, or 3) if it cannot fit in the categories proposed in this document** | | |
| --- | --- | --- |
| id | **Delivery identifier** – Provide the identifier used by your cohort to identify the delivery (Data type: char or numeric) | |
| N_preterm | **Number of prior preterm births** – Provide the number of preterm births the mother had prior to the index delivery of child. Use 0 if the index delivery is the first birth or the delivery mother did not have any prior preterm children. (Data type: numeric) | |
| region | **Cohort-reported census region of child birth location** – Provide the census region of the birth location for each child participant. (Data type: numeric)  1 = Northeast  2 = South  3 = Midwest  4 = West  5 = Puerto Rico  For more information about US census region, please see:  <https://www2.census.gov/geo/pdfs/maps-data/maps/reference/us_regdiv.pdf> | |
| address | **Address of primary residence during pregnancy** – Provide the earliest report of the primary residence address of the mother during pregnancy. Please concatenate the street number, street name, city, states, and zip code into a single field if they are saved in separate fields in your database. (Data type: char)  *Short Tips:*   - Make sure the zip code is 5 digits. Add a leading zero(s) if necessary. - Separate the different address components with a space. - Do not include any characters (periods, commas, etc.) in the address. - Addresses should be in proper order (3333 Burnet Ave Cincinnati OH 45229 NOT 3333 Burnet Ave Cincinnati 45229 OH) - Include street prefix or suffix if present (123 E Main St not 123 Main St.) - Spelling should be as accurate as possible, but the program does complete “fuzzy matching” so an exact match is not necessary. - Capitalization does not affect results. - Omit apartment numbers or “second address line”. - Apostrophes in street names are okay (O’Neal Street).   **Example:**  415 N Washington Street Baltimore MD 21231  [street number] [street name] [city] [state] [zip code]  *Please refer to Section 2.4, Section 2.5 and Appendix C in the EC0015 Cohort Data Compilation Instructions* for more details. | |
| GA_METH | **Method used for best estimate of gestational age at birth** – Please refer to *the* *hierarchy for methods* *of gestational age at birth* as below. There are eight hierarchies, with 1 being the most preferred (highest ranking) to 8 being less preferred (lowest ranking). Please provide **only one** gestational age at birth per index delivery of child that was obtained by the highest ranking method for that child in your cohort. (Data type: numeric)  *The* *hierarchy for methods* *of gestational age at birth:*  1 = estimated from crown rump length (CRL) of an early (<13 weeks 6 days) ultrasound, alone or confirming dating from a sure last menstrual period (LMP) date; or dating based on embryo placement date following in vitro fertilization; or dating based on artificial insemination date  2 = dating from an ultrasound taken in the second trimester (<28 weeks) with fetal biparietal diameter (BPD) dating within 2 weeks of sure LMP  3 = dating from an ultrasound taken in the second trimester (<28 weeks) with unsure LMP date or no LMP date  4 = report from obstetrical medical record reporting “consensus” estimated date of delivery (EDD) (i.e., combination of dates, scans, exam, etc.)  5 = dating by LMP with a third trimester ultrasound dating within 2 weeks of EDD by LMP dating  6 = dating from LMP date only  7 = reported by mother  8 = report from newborn medical record if examined by neonatologist and preterm | **Example:**  Delivery #0000 has three records of gestational age at birth. Each of them was assessed by a different method as below:  Record #1:  37 weeks 5 days, by CRL of an early ultrasound  Record #2: 36 weeks 2 days, by an ultrasound taken in the second trimester with no LMP date  Record #3: 38 weeks 0 days reported by mother  For Delivery #0000, only the Record #1 should be compiled in the EC0015 analytical dataset, with GA_METH=1  GA_WKS=37  GA_DAYS=5  Please use whole numbers for variable GA_WKS and GA_DAYS |
| GA_WKS | **Gestational age at birth (completed weeks)** -- Provide the best estimate available for the child’s gestational age at birth, based on *the hierarchy for methods of gestational age* determination  (Data type: integer \| Range: 22-43) |  |
| GA_DAYS | **Gestational age at birth (days)** -- Provide the best estimate available for the child’s gestational age at birth, based on *the hierarchy for methods of gestational age* determination  (Data type: integer \| Range: 0-6) |  |
| MAT_AGE | **Mother’s age at child’s birth** – Provide the age of the biological mother at child’s birth. You may provide decimal values. (Data type: numeric \| Range: 10–60 years) | |
| MAT_RACE | **Maternal race** – Provide the primary race of the biological mother (Data type: numeric)  1 = White  2 = Black  3 = Asian  4 = Native Hawaiian or other Pacific Islander  5 = American Indian or Alaska Native  6 = Multiple race  7 = Other race | |
| MAT_ETHNIC | **Maternal ethnicity** – Provide the primary ethnicity of the biological mother (Data type: numeric)  0 = Not Hispanic or Latino  1 = Hispanic or Latino | |
| MAT_JOB | **Maternal employment** – Provide the prenatal employment of the biological mother at the earliest prenatal time point available (Data type: numeric)  0 = Not employed  1 = Employed | |
| MAT_EDUC | **Maternal education** – Provide the highest level of education the biological mother had at the earliest prenatal time point available using the following categories (Data type: numeric)  1 = Less than high school  2 = High school diploma or a GED (or equivalent)  3 = Some college (includes associates, vocational, technical)  4 = Bachelor's Degree  5 = Graduate or Professional degree | |
| MAT_cEDUC | **Maternal education (alternative coding)** – Provide the highest level of education the biological mother had at the earliest prenatal time point available using the following categories (Data type: numeric)  1 = high school graduate or less  2 = some college or above  This field should only be populated if unable to fit in the categories of MAT_EDUC | |
| PN_HH_INC | **Prenatal household income (dollars per year)** – Provide the annual household income in US dollars at the earliest prenatal time point available using the following categories (Data type: numeric)  1 = Under $25,000  2 = $25,000 - $49,999  3 = $50,000 - $74,999  4 = $75,000 - $99,999  5 = $100,000 and above | |
| PN_HH_cINC | **Prenatal household income (dollars per year, alternative coding)** – Provide the annual household income in US dollars at the earliest prenatal time point available using the following categories (Data type: numeric)  0 = Under $100,000  1 = $100,000 and above  This field should only be populated if unable to fit in the categories of PN_HH_INC | |
| HH_COUNT | **Household size** – Provide the number of individuals living in primary residential household (excluding the index child) at the earliest prenatal time point available (Data type: integer \| Range: 1-25) | |
| PN_HH_INCLEV | **Prenatal household income (%FPL)** – Provide the annual household income according to percent of the Federal Poverty Guideline issued by the Department of Health & Human Services at the earliest prenatal time point available (Data type: numeric)  1 = Less than 100% of the federal poverty level  2 = 100 - 199% of the federal poverty level  3 = 200-299% of the federal poverty level  4 = 300-399% of the federal poverty level  5 = 400% of the federal poverty level or higher | |
| INSUR | **Prenatal health insurance** – Provide the maternal prenatal health insurance status (Data type: numeric)  0 = No prenatal health insurance  1 = Prenatal health insurance | |
| INSUR_PUBLIC | **Prenatal health insurance type: public** – Provide the maternal prenatal health insurance type (Data type: numeric)  0 = No public health insurance during the prenatal period  1 = Public health insurance during the prenatal period (Medicaid or Medicare) | Both public and private health insurance can be selected if the mother has both during her pregnancy; neither can be selected if she has neither during her pregnancy. |
| INSUR_PRIVATE | **Prenatal health insurance type: private**– Provide the maternal prenatal health insurance type (Data type: numeric)  0 = No private health insurance during the prenatal period  1 = Private health insurance during the prenatal period |  |
| PAT_AGE | **Father’s age at child’s birth** – You may provide decimal values.  (Data type: numeric \| Range: 10–60 years) | |
| PAT_RACE | **Paternal race** – Provide the primary race of the father (Data type: numeric)  1 = White  2 = Black  3 = Asian  4 = Native Hawaiian or other Pacific Islander  5 = American Indian or Alaska Native  6 = Multiple race  7 = Other race | |
| PAT_ETHNIC | **Paternal ethnicity** – Provide the primary ethnicity of the father (Data type: numeric)  0 = Not Hispanic or Latino  1 = Hispanic or Latino | |
| PAT_JOB | **Paternal employment** – Provide the Paternal prenatal employment at the earliest prenatal time point available (Data type: numeric)  0 = Not employed  1 = Employed | |
| PAT_EDUC | **Paternal education** – Provide the highest level of education the father had at the earliest prenatal time point available using the following categories (Data type: numeric)  1 = Less than high school  2 = High school diploma or a GED (or equivalent)  3 = Some college (includes associates, vocational, technical)  4 = Bachelor's Degree  5 = Graduate or Professional degree | |
| PAT_cEDUC | **Paternal education (alternative coding)** – Provide the highest level of education the father had at the earliest prenatal time point available using the following categories (Data type: numeric)  1 = high school graduate or less  2 = some college or above  This field should only be populated if unable to fit in the categories of PAT_EDUC | |
| DOB_M | **Month of birth of child**  (Data type: integer \| Range: 1-12) | |
| DOB_Y | **Year of birth of child**  (Data type: integer \| Range: 1980-2017) | |
| SEX | **Child biological sex at birth**  (Data type: numeric)  1 = male  2 = female | |
| CONC_ASSIST | **Conception resulting from assisted reproductive technology**  (Data type: numeric)  0 = natural conception  1 = conception by assisted reproductive technology | |
| PN_MAT_TBK | **Maternal prenatal tobacco use during the index pregnancy** – Use may occur at any point during the pregnancy (Data type: numeric)  0 = no known use during the pregnancy  1 = known use during the pregnancy | |
| PN_MAT_ALCO | **Maternal prenatal alcohol use during the index pregnancy** – Use may occur at any point during the pregnancy (Data type: numeric)  0 = no known use during the pregnancy  1 = known use during the pregnancy | |
| PN_MAT_DRUG | **Maternal prenatal recreational drug use during the index pregnancy** – Recreational drug use is defined use of any drug (whether an illicit drug, prescription medication, or prescription medication) without medical justification. Use may occur at any point during the index pregnancy (Data type: numeric)  0 = no known use during the pregnancy  1 = known use during the pregnancy | |
| PN_MAT_CANN | **Maternal marijuana, cannabis, or cannabinoid use during in the index pregnancy**  (Data type: numeric)  0 = no known use during the pregnancy  1 = known medical use during the pregnancy  2 = known recreational (non-medical) use during the pregnancy  3 = known use, but unknown if medical or recreational | Medical use is defined as use as prescribed by a health care provider (such as might occur for chronic pain or opiate dependency or opiate use disorder).  Recreational use is defined as use without medical justification. Use may occur at any point during the index pregnancy |
| PN_MAT_STIM | **Maternal stimulant drug use (such as methamphetamines/amphetamines, cocaine, Adderall or Ritalin) in the index pregnancy**  (Data type: numeric)  0 = no known use during the pregnancy  1 = known medical use during the pregnancy  2 = known recreational (non-medical) use during the pregnancy  3 = known use, but unknown if medical or recreational |  |
| PN_MAT_OPIATES | **Maternal opiate drug use (such as heroin, methadone, buprenorphine, opiate pain medications) in the index pregnancy other than during labor**  (Data type: numeric)  0 = no known use during the pregnancy  1 = known medical use during the pregnancy  2 = known recreational (non-medical) use during the pregnancy  3 = known use, but unknown if medical or recreational |  |
| PN_CARE | **Prenatal care receipt** (Data type: numeric)  0 = no prenatal care received  1 = prenatal care received | |
| PN_INFECT_UTI | **Occurrence of urinary tract infections in the index pregnancy** – For purposes of this analysis, urinary tract infections include any of the following: asymptomatic bacteriuria, cystitis or lower tract infection, pyelonephritis or upper tract infection  (Data type: numeric)  0 = no  1 = yes | |
| PN_INFECT_RTRACT | **Occurrence of reproductive tract infections in the index pregnancy** – For purposes of this analysis, reproductive tract infections include any of the following: bacterial vaginosis, chlamydia, gonorrhea, trichomoniasis, cervicitis, pelvic inflammatory disease (Data type: numeric)  0 = no  1 = yes | |
| PARITY | **Parity** – Provide the parity, or the number of births that the mother had after 20 weeks’ gestation (excluding index child). Twin or higher order births count as a single birth event for determining parity  (Data type: integer \| Range: 0-25) | |
| MAT_HT_CM | **Maternal height (cm)**  (Data type: numeric \| Range: 50-250) | |
| MAT_HT_METH | **Method of determining maternal height** (Data type: numeric)  1 = measured by researcher  2 = abstracted from the medical record  3 = maternal self-report | |
| MAT_WT_KG | **Maternal weight (kg)** – Provide the maternal weight during pregnancy at the earliest prenatal point available  (Data type: numeric \| Range: 20-250) | |
| MAT_WT_METH | **Method of determining maternal weight** (Data type: numeric)  1 = measured by researcher  2 = abstracted from the medical record  3 = maternal self-report | |
| MAT_WT_TIME | **Timing of maternal weight measurement** – Provide the timing of the earliest maternal prenatal weight measurement or determination  (Data type: numeric)  1 = 12 weeks’ gestation or less  2 = 13 through 19 weeks’ gestation  3 = 20 through 27 weeks’ gestation  4 = 28 weeks gestation or greater | |
| PN_MARITAL | **Prenatal marital status of mother** (Data type: numeric)  1 = married  2 = widowed/separated/divorced  3 = single | |
| MAT_COUNTRY | **Maternal country of origin** (Data type: numeric)  0 = US  1 = Mexico  2 = Korea  3 = India  4 = Germany  5 = El Salvador  6 = Cuba  7 = China  8 = Canada  9 = Vietnam  10 = Philippines  11= Other | |
| PRETERM_HIST | **Maternal history of any prior preterm birth in prior pregnancies** (Data type: numeric)  0 = no  1 = yes | |
| PROM | **Premature rupture of the membranes in index pregnancy**  (Data type: numeric)  0 = no  1 = yes | |
| PREECLAMPSI | **Occurrence of preeclampsia in index pregnancy**  (Data type: numeric)  0 = no  1 = yes | |
| GEST_HTN | **Occurrence of gestational hypertension in index pregnancy** -- Gestational hypertension is defined as hypertension that develops after 20 weeks’ gestation in the index pregnancy (Data type: numeric)  0 = no  1 = yes | |
| GEST_DIAB | **Occurrence of gestational diabetes in index pregnancy** -- Gestational diabetes diagnosed during the index pregnancy  (Data type: numeric)  0 = no  1 = yes | |
| IUGR | **Occurrence of intrauterine growth restriction in the index pregnancy** (Data type: numeric)  0 = no  1 = yes | |
| PLAC_ABRUPT | **Occurrence of placental abruption in the index pregnancy**  (Data type: numeric)  0 = no  1 = yes | |
| PLAC_PREV | **Occurrence of placental previa in the index pregnancy**  (Data type: numeric)  0 = no  1 = yes | |
| CHRON_HTN | **Presence of chronic hypertension in the biological mother** -- Chronic hypertension is defined as hypertension that occurs prior to the index pregnancy or before 20 weeks’ gestation within the index pregnancy (Data type: numeric)  0 = no  1 = yes | |
| CHRON_DM | **Presence of chronic type 1 and/or type 2 diabetes mellitus in the biological mother** -- Chronic diabetes is defined as diabetes that developed prior to the index pregnancy (Data type: numeric)  0 = no  1 = yes | |
| CHRON_OTHER | **Diagnosis of any other chronic conditions prior to the index pregnancy** – Provide the diagnosis of any other chronic conditions (excluding hypertension and diabetes) among following: asthma, other lung disease, cardiac disease other than hypertension, autoimmune/connective tissue disease, renal disease, thyroid disease (Data type: numeric)  0 = no  1 = yes | |
| CHRON_INFECT | **Presence of chronic infections in the biological mother before or during pregnancy –** For purposes of this analysis, chronic infections including HIV, Hepatitis B, and Hepatitis C (Data type: numeric)  0 = no  1= yes | |
| LABOR_TYPE | **Types of labor for index pregnancy** (Data type: numeric)  1 = spontaneous  2 = induced  3 = C-section without spontaneous or induced labor | |
| DELIVERY_TYPE | **Types of delivery for index pregnancy** (Data type: numeric)  1 = vaginal  2 = C-section | |
| SINGLE | **Singleton birth** – Provide the singleton birth of child. If there is not an existing variable regarding singleton birth in your database, please also review your study materials and see if you can find any information regarding this variable (Data type: numeric)  0 = Multiple (twins, triplets, or higher order)  1 = singleton | |
| CONGEN_ANOM | **Congenital anomalies** – Provide the presence of structural and/or functional anomalies such as neural tube defects/spina bifida, cleft lip or cleft palate, Down syndrome or other chromosomal abnormalities, congenital heart disease, congenital limb defect, cystic fibrosis, etc.  (Data type: numeric)  0 = no  1 = yes | |
| STILLBIRTH | **Still birth –** Still birth is defined as intrauterine fetal death after 20 weeks. If there is not an existing variable regarding still birth in your database, please also review your study materials and see if you can find any information regarding this variable (Data type: numeric)  0 = live born  1 = still born | |
