# Supplementary material for: Racial and geographic variation in effects of maternal education and neighborhood-level measures of socioeconomic status on gestational age at birth: Findings from the ECHO cohorts
Source: PLoS One. 2021 Jan 8;16(1):e0245064. doi: 10.1371/journal.pone.0245064 (PMC7794036; doi:10.1371/journal.pone.0245064)
Supplement: S2 Table — (DOCX) [file pone.0245064.s002.docx]

**S2 Table**. Race and ethnicity frequencies of mothers of singleton live births by gestational age at birth category

| **Variable** | **TOTAL SAMPLE**  **N=25,526 (column %)** | **Preterm birth**  **(22 - <37 weeks)**  **n = 1,864 (7.3%)** | **Early term birth**  **(37- <39 weeks)**  **n = 6,191 (24.3%)** | **Full term birth**  **(39 - <41 weeks)**  **n = 14,813 (58.0%)** | **Late or post-term birth**  **(41- 43 weeks)**  **n = 2,658 (10.4%)** |
| --- | --- | --- | --- | --- | --- |
| **Maternal race** | | | | | |
| White | 14140 (55.39%) | 833 (44.69%) | 3148 (50.85%) | 8436 (56.95%) | 1723 (64.82%) |
| Black | 3832 (15.01%) | 403 (21.62%) | 1022 (16.51%) | 2094 (14.14%) | 313 (11.78%) |
| Asian | 1852 (7.26%) | 152 (8.15%) | 530 (8.56%) | 1045 (7.05%) | 125 (4.7%) |
| Native Hawaiian or Other Pacific Islander | 84 (0.33%) | 6 (0.32%) | 19 (0.31%) | 52 (0.35%) | 7 (0.26%) |
| American Indian or Alaska Native | 1431 (5.61%) | 140 (7.51%) | 445 (7.19%) | 726 (4.9%) | 120 (4.51%) |
| Multiple race | 681 (2.67%) | 46 (2.47%) | 156 (2.52%) | 400 (2.7%) | 79 (2.97%) |
| Other Race | 1222 (4.79%) | 121 (6.49%) | 282 (4.55%) | 709 (4.79%) | 110 (4.14%) |
| Missing | 2284 (8.95%) | 163 (8.74%) | 589 (9.51%) | 1351 (9.12%) | 181 (6.81%) |
| **Maternal ethnicity** | | | | | |
| Hispanic or Latino | 4813 (18.86%) | 385 (20.65%) | 1186 (19.16%) | 2804 (18.93%) | 438 (16.48%) |
| Non-Hispanic or Latino | 19958 (78.19%) | 1431 (76.77%) | 4806 (77.63%) | 11549 (77.97%) | 2172 (81.72%) |
| Missing | 755 (2.96%) | 48 (2.58%) | 199 (3.21%) | 460 (3.11%) | 48 (1.81%) |
