# Supplementary material for: Racial and geographic variation in effects of maternal education and neighborhood-level measures of socioeconomic status on gestational age at birth: Findings from the ECHO cohorts
Source: PLoS One. 2021 Jan 8;16(1):e0245064. doi: 10.1371/journal.pone.0245064 (PMC7794036; doi:10.1371/journal.pone.0245064)
Supplement: S3 Table — (DOCX) [file pone.0245064.s003.docx]

**S3 Table**. Maternal education, neighborhood-level socioeconomic status, and adjusted odds of preterm, early term, and late-post term births compared to full term births by maternal race/ethnicity^1^

|  | **Preterm**  **Adjusted OR (95% CI)** | | | | | **Early-term**  **Adjusted OR (95% CI)** | | | | | **Late- or post-term**  **Adjusted OR (95% CI)** | | | | |
| --- | --- | --- | --- | --- | --- | --- | --- | --- | --- | --- | --- | --- | --- | --- | --- |
|  | **Overall** | **NH White** | **NH Black** | **NH other race** | **Hispanic** | **Overall** | **NH White** | **NH Black** | **NH other race** | **Hispanic** | **Overall** | **NH White** | **NH Black** | **NH other race** | **Hispanic** |
| **Prenatal Maternal Education^1^** | | | | | | | | | | | | | | | |
| Bachelor's or above | 0.88 (0.65-1.19) | 0.82 (0.57-1.17) | 0.58 (0.19-1.77) | —^2^ | —^2^ | 0.89 (0.76-1.06) | 0.86 (0.70-1.04) | 0.99 (0.60-1.63) | 1.66 (0.82-3.39) | —^2^ | 1.11 (0.85-1.45) | 1.04 (0.77-1.39) | —^2^ | 0.98 (0.31-3.14) | —^2^ |
| Some College | ref | ref | ref | ref | ref | ref | ref | ref | ref | ref | ref | ref | ref | ref | ref |
| High school or less | 1.18 (0.90-1.56) | 1.06 (0.66-1.70) | 1.02 (0.59-1.76) | 1.52 (0.88-2.64) | —^2^ | 1.13 (0.96- 1.34) | 1.07 (0.83-1.38) | 1.19 (0.71-2.00) | 1.26 (0.91-1.75) | 0.74 (0.27-2.07) | 1.23 (0.93- 1.64) | 1.11 (0.73-1.67) | 1.05 (0.50-2.20) | 1.36 (0.47-3.93) | —^2^ |
| **Census tract – Urbanicity^3^** | | | | | | | | | | | | | | | |
| Rural | 1.31 (0.91- 1.88) | 1.56 (0.98-2.46) | —^2^ | 1.22 (0.62-2.41) | —^2^ | 1.03 (0.85- 1.25) | 0.98 (0.78-1.23) | 0.96 (0.43-2.15) | 1.13 (0.75-1.7) | 1.38 (0.42-4.52) | 0.89 (0.63- 1.25) | 0.72 (0.5-1.04) | —^2^ | 1.48 (0.72-3.01) | 1.93 (0.26-14.43) |
| Urban | ref | ref | ref | ref | ref | ref | ref | ref | ref | ref | ref | ref | ref | ref | ref |
| **Census tract – Percent Black^4^** | | | | | | | | | | | | | | | |
| % Black above | 0.80 (0.47- 1.38) | 0.94 (0.31-2.82) | —^2^ | —^2^ | —^2^ | 1.00 (0.76- 1.32) | 0.93 (0.65-1.33) | —^2^ | 0.93 (0.51-1.68) | 0.81 (0.20-3.21) | 0.82 (0.51- 1.34) | 1.3 (0.21-8.23) | —^2^ | —^2^ | — |
| % black below | ref | ref | ref | ref | ref | ref | ref | ref | ref | ref | ref | ref | ref | ref | ref |
| **Census tract – Percent Poverty^5^** | | | | | | | | | | | | | | | |
| % Poverty above | 0.92 (0.65- 1.31) | 1.15 (0.73-1.80) | 0.49 (0.27-0.88) | 0.96 (0.45-2.05) | —^2^ | 1.13 (0.96- 1.33) | 1.17 (0.84-1.64) | 1.00 (0.67-1.48) | 0.90 (0.59-1.38) | 1.52 (0.30-7.83) | 1.23 (0.90- 1.69) | 1.36 (0.97-1.91) | 1 (0.31-3.19) | 1.15 (0.56-2.37) | 1.27 (0.21-7.60) |
| % Poverty below | ref | ref | ref | ref | ref | ref | ref | ref | ref | ref | ref | ref | ref | ref | ref |

Abbreviations: CI = confidence interval; NH = Non-Hispanic; OR = odds ratio; ref = reference.

^1^ Multinomial logistic regression adjusted for census tract urbanicity, census tract-percent black, census tract-percent poverty, maternal age, parity, marital status, child sex, chronic health conditions, prenatal substance use, prenatal cardiometabolic complications, obstetrical complications, prenatal maternal body mass index, private insurance status, chronic infections, prenatal infections, history of preterm birth.

^2^ Meta-analysis was unable to be performed due to unstable cohort-specific estimates.

^3^ Multinomial logistic regression adjusted for prenatal maternal education, census tract-percent black, census tract-percent poverty, maternal age, parity, marital status, child sex, chronic health conditions, prenatal substance use, prenatal cardiometabolic complications, obstetrical complications, prenatal maternal body mass index, private insurance status, chronic infections, prenatal infections, history of preterm birth.

^4^ Multinomial logistic regression adjusted for prenatal maternal education, census-tract urbanicity, census tract-percent poverty, maternal age, parity, marital status, child sex, chronic health conditions, prenatal substance use, prenatal cardiometabolic complications, obstetrical complications, prenatal maternal body mass index, private insurance status, chronic infections, prenatal infections, history of preterm birth.

^5^ Multinomial logistic regression adjusted for prenatal maternal education, census tract urbanicity, census tract-percent black, maternal age, parity, marital status, child sex, chronic health conditions, prenatal substance use, prenatal cardiometabolic complications, obstetrical complications, prenatal maternal body mass index, private insurance status, chronic infections, prenatal infections, history of preterm birth.
