# Supplementary material for: Racial and geographic variation in effects of maternal education and neighborhood-level measures of socioeconomic status on gestational age at birth: Findings from the ECHO cohorts
Source: PLoS One. 2021 Jan 8;16(1):e0245064. doi: 10.1371/journal.pone.0245064 (PMC7794036; doi:10.1371/journal.pone.0245064)
Supplement: S1 Ethics — (DOCX) [file pone.0245064.s005.docx]

**S1 Ethics. ECHO Awardees and Cohorts that contributed aggregate data results for this analysis:**

| Cohort ID | Cohort Name | Cohort Contact PI | Institutional Review Board (IRB) | IRB Approval Number |  |
| --- | --- | --- | --- | --- | --- |
| 10101 | ECHO in Puerto Rico | Akram Alshawabkeh | WIRB | 20181210 |  |
| 10401 | 35th Multicenter Airway Research Collaboration | Carlos Camargo | Partners | 2016P001836 |  |
| 10402 | 43rd Multicenter Airway Research Collaboration | Carlos Camargo | Partners | 2016P002882 |  |
| 10601 | Healthy Start | Dana Dabelea | Western IRB (WIRB) | 20181210 |  |
| 10801 | Boricua Youth Study | Cristiane Duarte | New York State Psychiatric Institute | 7377 |  |
| 10901 | Atlanta ECHO Cohort of Emory University | Anne Dunlop | Emory University | 91254 |  |
| 11001 | Safe Passage Study | Amy Elliott | WIRB  Avera IRB | \| 1260729 \| \| --- \| \| \| 2017.091 / 100475 \| \| --- \| \| |  |
| 11201 | PETALS | Assiamira Ferrara | WIRB | 20181210 |  |
| 11202 | KPRB | Assiamira Ferrara | WIRB | 20181210 |  |
| 11303 | Tucson Children's Respiratory Study | James Gern | University of Arizona IRB | 7500000005 |  |
| 11304 | Tucson Infant Immune Study | James Gern | University of Arizona IRB | 9600000328A017 |  |
| 11305 | Wisconsin Infant Study Cohort | James Gern | MCRI IRB | KEI10613 |  |
| 11306 | Childhood Origins of Asthma Study | James Gern | Health Sciences IRB | 2017-0306 |  |
| 11307 | Urban Environment and Childhood Asthma | James Gern | 1. Washington University in St. Louis IRB  2. Johns Hopkins  3. Columbia IRB  4. Boston University | 1. 201702039  2. NA_00044286/CIR00026928  NA_00025027/CIR00028419  NA_00070888/CIR00028423  3. AAAC5139  4. H-23320 |  |
| 11309 | Infant Susceptibility to Pulmonary Infections and Asthma Following RSV Exposure | James Gern | Vanderbilt University IRB | 111299 |  |
| 11310 | Epidemiology of Home Allergens and Asthma Study | James Gern | Partners | 2017P000073 |  |
| 11311 | Wayne County Health Environment Allergy and Asthma | James Gern | Henry Ford Health System | 1881 |  |
| 11312 | Childhood Allergy/Asthma Study | James Gern | Henry Ford Health System | 2855 |  |
| 11401 | MADRES | Frank Gilliland | University of Southern California | HS-15-00498 |  |
| 11601 | ReCHARGE: Revisiting CHildhood Autism Risks from Genes and the Environment Study | Irva Hertz-Picciotto | University of California Davis | 952089 |  |
| 11701 | Pittsburgh Girls Study | Alison Hipwell | University of Pittsburgh | PRO17050429 |  |
| 11801 | New Hampshire Birth Cohort Study | Margaret Karagas | Dartmouth College | CPHS20844 |  |
| 11901 | CANDLE | Catherine Karr | University of Washington | 0638 |  |
| 11902 | The Infant Development and Environment II Study | Catherine Karr | University of Washington | 0638 |  |
| 11903 | GAPPS | Catherine Karr | University of Washington | 0638 |  |
| 12101 | Early Growth and Development Study | Leslie Leve | University of Oregon | 08082016.007 |  |
| 12102 | Early Growth and Development Study - Cohort II | Leslie Leve | University of Oregon | 08082016.007 |  |
| 12103 | Early Parenting of Children | Leslie Leve | University of Oregon | 08082016.007 |  |
| 12201 | Understanding Risk Gradients from Environment on Native American Child Health Trajectories: Toxicants, Immunomodulation, Metabolic syndromes, & Metals Exposure | Johnnye Lewis | University of New Mexico Health Sciences Center  IRB Navajo Nation Human Research Review Board | (#19-423 and 11-310)  (NNR#19.360 and 11.323) |  |
| 12301 | VDAART | Augusto Litonjua | Brigham & Women’s Hospital | 2009P000557 |  |
| 12401 | Vitamin C to Decrease Effects of Smoking in Pregnancy on Infant Lung Function | Cynthia McEvoy | Oregon Health & Science University | 6091 |  |
| 12402 | In-Utero Smoke, Vitamin C, and Newborn Lung Function | Cynthia McEvoy | Oregon Health & Science University | 6091 |  |
| 12501 | Kennedy Krieger - Baby Siblings Research Consortium | Craig Newschaffer | Johns Hopkins Medicine IRB | NA_00038069 / CIR00023036 |  |
| 12502 | University of California Davis - Baby Siblings Research Consortium | Craig Newschaffer | UCDavis IRB | 243919-28 |  |
| 12503 | Autism Spectrum Disorders-Enriched Risk - University of  Washington - BRSC | Craig Newschaffer | University of Washington IRB | 00000499 |  |
| 12504 | Autism Spectrum Disorders-Enriched Risk-BRSC Universtiy of  Miami | Craig Newschaffer | University of Miami IRB | 20160954 |  |
| 12505 | University of Washington-Infant Brain Imaging Study | Stephen Dager | University of Washington IRB | 50975 |  |
| 12506 | Autism Spectrum Disorders - Enriched Risk - IBIS -  Washington University, St. Louis | Craig Newschaffer | Washington University IRB | 201611161 |  |
| 12507 | Infant Brain Imaging Study | Robert Schultz | The Children’s Hospital of Philadelphia Research Institute IRB | 07-005689 |  |
| 12508 | Infant Brain Imaging Study | Joseph Piven | University of North Carolina-Chapel Hill | 05-2293 |  |
| 12509 | Early Autism Risk Longitudinal Investigation | Heather Volk | Johns Hopkins Bloomberg School of Public Health IRB | 00002032 |  |
| 12510 | Early Autism Risk Longitudinal Investigation | Rebecca Schmidt | ECHO WIRB IRB  UCD IRB for ASD-ER  UCD IRB for EARLI | 20181210  960711 214753-32 |  |
| 12511 | Early Autism Risk Longitudinal Investigation | Lisa Croen | WIRB | 20181210 |  |
| 12512 | Autism Spectrum Disorders - Enriched Risk EARLI - Drexel  University | Craig Newschaffer | Drexel University IRB | 1609004845  1710005749 |  |
| 12513 | University of California - Markers of Autism Risk in Babies | Craig Newschaffer | ECHO WIRB IRB  UCD IRB for ASD-ER  UCD IRB for EARLI | 20181210  960711 214753-32 |  |
| 12601 | Rochester | Tom O'Connor | University of Rochester | 58456 |  |
| 12701 | Project Viva | Emily Oken | Harvard Pilgrim Healthcare | 235301 |  |
| 12901 | ARCH | Nigel Paneth | Michigan State University  Michigan Department of Health and Human Services | LEGACYC07-120  201312-03-FC |  |
| 13001 | Mothers and Newborns | Frederica Perera | Columbia University Medical Center | AAAA6110 |  |
| 13101 | Illinois Kids Development Study | Susan Schantz | University of Illinois Urbana Champaign | 09498 |  |
| 13102 | Chemicals in our Bodies | Susan Schantz | University of California, San Francisco | UCSF IRB #  13-12160 |  |
| 13201 | Utah's Children's Project | Joseph Stanford | University of Utah | 63183 |  |
| 13301 | The NYU Children’s Health and Environment Study | Leonardo Trasande | NYU Grossman School of Medicine | s15-00778, s16-01617 |  |
| 13501 | Asthma Coalition on Community, Environment & Stress | Rosalind Wright | Program for the Protection of Human Subjects at Mount Sinai | 12-00661 |  |
| 13502 | Programming of Intergenerational Stress Mechanisms | Rosalind Wright | Program for the Protection of Human Subjects at Mount Sinai | 12-00875 |  |
| 13503 | Inova Childhood Longitudinal Study | Rosalind Wright | Inova | U19-02-3423 |  |
