# Supplementary material for: Racial and geographic variation in effects of maternal education and neighborhood-level measures of socioeconomic status on gestational age at birth: Findings from the ECHO cohorts
Source: PLoS One. 2021 Jan 8;16(1):e0245064. doi: 10.1371/journal.pone.0245064 (PMC7794036; doi:10.1371/journal.pone.0245064)
Supplement: S1 Funding — (DOCX) [file pone.0245064.s006.docx]

**S1 Funding. Grant numbers for ECHO Awardees and Cohorts that contributed aggregate data for this analysis:**

| Cohort ID | Cohort Name | Cohort Contact PI /Manuscript Author | NIH Grant Number |
| --- | --- | --- | --- |
| 10101 | ECHO in Puerto Rico | Akram Alshawabkeh | UH3OD023251 |
| 10401 | 35^th^ Multicenter airway Research Collaboration | Carlos Camargo | UH3OD023253 |
| 10402 | 43^rd^ Multicenter Airway Research Collaboration | Carlos Camargo | UH3OD023253 |
| 10601 | Healthy Start | Dana Dabelea | UH3OD023248 |
| 10801 | Boricua Youth Study | Cristiane Duarte | UH3OD023328 |
| 10901 | Atlanta ECHO Cohort of Emory University | Anne Dunlop | UH3 OD023318 |
| 11001 | Safe Passage Study | Amy Elliott | UH3OD023279 |
| 11201 | PETALS | Assiamira Ferrara /Lyndsay Alvalos | UH3OD023289 |
| 11202 | KPRB | Assiamira Ferrara /Lyndsay Alvalos | UH3OD023289 |
| 11303 | Tucson Children’s Respiratory Study | James Gern | UH3OD023282 |
| 11304 | Tucson Infant Immune Study | James Gern | UH3OD023282 |
| 11305 | Wisconsin Infant Study Cohort | James Gern | UH3OD023282 |
| 11306 | Childhood Origins of Asthma Study | James Gern | UH3OD023282 |
| 11307 | Urban Environment and Childhood Asthma | James Gern | UH3OD023282 |
| 11309 | Infant Susceptibility to Pulmonary Infections and Asthma Following RSV Exposure | James Gern | UH3OD023282 |
| 11310 | Epidemiology of Home Allergens and Asthma Study | James Gern | UH3OD023282 |
| 11311 | Wayne County Health Environment Allergy and Asthma | James Gern | UH3OD023282 |
| 11312 | Childhood Allergy/Asthma Study | James Gern | UH3OD023282 |
| 11401 | MADRES | Frank Gilliland /Carrie Breton | UH3OD023287 |
| 11601 | ReCHARGE: Revisiting Childhood Autism Risks from Genes and the Environment Study | Irva Hertz-Picciotto /Yunin Ludena-Rodriguez | UH3OD023365 |
| 11701 | Pittsburgh Girls Study | Alison Hipwell | UH3OD023244 |
| 11801 | New Hampshire Birth Cohort Study | Margaret Karagas | UH3OD023275 |
| 11901 | CANDLE | Catherine Karr /Sheela Sathyanarayana | UH3OD023271 |
| 11902 | The Infant Development and the Environment II Study | Catherine Karr /Sheela Sathyanarayana | UH3OD023271 |
| 11903 | GAPPS | Catherine Karr /Sheela Sathyanarayana | UH3OD023271 |
| 12101 | Early Growth and Development Study | Leslie Leve | UH3OD023389 |
| 12102 | Early Growth and Development Study – Cohort II | Leslie Leve | UH3OD023389 |
| 12103 | Early Parenting of Children | Leslie Leve | UH3OD023389 |
| 12201 | Understanding Risk Gradients from Environment on Native American Child Health Trajectories: Toxicants, Immunomodulation, Metabolic syndromes, & Metals Exposure | Johnnye Lewis | UH3OD023344 |
| 12301 | VDAART | Augusto Litonjua | UH3OD023268 |
| 12401 | Vitamin C to Decrease Effects of Smoking in Pregnancy yon Infant Lunch Function | Cynthia McEvoy | UH3OD023288 |
| 12402 | In-Utero Smoke, Vitamin C, and Newborn Lunch Function | Cynthia McEvoy | UH3OD023288 |
| 12501 | Kennedy Krieger – Baby Siblings Research Consortium | Craig Newschaffer | UH3OD023342 |
| 12502 | University of California Davis - Baby Siblings Research  Consortium | Craig Newschaffer | UH3OD023342 |
| 12503 | Autism Spectrum Disorders-Enriched Risk - University of  Washington - BRSC | Craig Newschaffer | UH3OD023342 |
| 12504 | Autism Spectrum Disorders-Enriched Risk-BRSC University of  Miami | Craig Newschaffer | UH3OD023342 |
| 12505 | University of Washington-Infant Brain Imaging Study | Stephen Dager | UH3OD023342 |
| 12506 | Autism Spectrum Disorders – Enriched Risk – IBIS – University of Washington, St. Louis | Craig Newschaffer | UH3OD023342 |
| 12507 | Infant Brain Imaging Study | Robert Schultz | UH3OD023342 |
| 12508 | Infant Brain Imaging Study | Joseph Piven | UH3OD023342 |
| 12509 | Early Autism Risk Longitudinal Investigation | Heather Volk | UH3OD023342 |
| 12510 | Early Autism Risk Longitudinal Investigation | Rebecca Schmidt | UH3OD023342 |
| 12511 | Early Autism Risk Longitudinal Investigation | Lisa Croen | UH3OD023342 |
| 12512 | Autism Spectrum Disorders – Enriched Risk EARLI – Drexel University | Craig Newschaffer | UH3OD023342 |
| 12513 | University of California – Markers of Autism Risk in Babies | Craig Newschaffer | UH3OD023342 |
| 12601 | Rochester | Thomas O’Connor | UH3OD023349 |
| 12701 | Project Viva | Emily Oken /Ken Kleinman | UH3OD023286 |
| 12901 | ARCH | Nigel Paneth/ Alicynne Glazier Essalmi | UH3OD023285 |
| 13001 | Mothers and Newborns | Frederica Perera | UH3OD023290 |
| 13101 | Illinois Kids Development Study | Susan Schantz | UH3OD023272 |
| 13102 | Chemicals in Our Bodies | Susan Schantz / Amy Padula, Elizabeth Hom Thepaksorn | UH3OD023272 |
| 13201 | Utah’s Children’s Project | Joseph Stanford | UH3OD023249 |
| 13301 | Safe Passage Study | Leonardo Trasande | UH3OD023305 |
| 13501 | Asthma Coalition on Community, Environment & Stress | Rosalind Wright /Whitney Cowell, Kathi Huddleston | UH3OD023337 |
| 13502 | Programming of Intergenerational Stress Mechanisms | Rosalind Wright /Whitney Cowell, Kathi Huddleston | UH3OD023337 |
| 13503 | Inova Childhood Longitudinal Study | Rosalind Wright | UH3OD023337 |

This research was also supported in part by the NIH for the Environmental Influences of Child Health Outcomes (ECHO) Data Analysis Center (U24OD023382).
