# Supplementary material for: Racial and geographic variation in effects of maternal education and neighborhood-level measures of socioeconomic status on gestational age at birth: Findings from the ECHO cohorts
Source: PLoS One. 2021 Jan 8;16(1):e0245064. doi: 10.1371/journal.pone.0245064 (PMC7794036; doi:10.1371/journal.pone.0245064)
Supplement: Attachment — Submitted filename: Response to Review, updated.docx [file pone.0245064.s007.docx]

PONE-D-20-14238R1
Racial and Geographic Variation in Effects of Maternal Education and Neighborhood-level Measures of Socioeconomic Status on Gestational Age at Birth: Findings from The ECHO Cohorts
PLOS ONE - **Author Response to Review**

Author Response to Editor’s Requests

1. Please amend your current ethics statement to include the full name of the ethics committee/institutional review board(s) that approved your specific study.

***Response:*** *We have amended our ethics statement to include reference to a Supplementary File (S1 Ethics) that provides the name of each Institutional Review Board (and IRB approval number) reviewing and approving the research of participating cohorts.*

**ECHO Awardees and Cohorts that contributed aggregate data results for this analysis:**

| Cohort ID | Cohort Name | Cohort Contact PI | Institutional Review Board (IRB) | IRB Approval Number |  |
| --- | --- | --- | --- | --- | --- |
| 10101 | ECHO in Puerto Rico | Akram Alshawabkeh | WIRB | 20181210 |  |
| 10401 | 35th Multicenter Airway Research Collaboration | Carlos Camargo | Partners | 2016P001836 |  |
| 10402 | 43rd Multicenter Airway Research Collaboration | Carlos Camargo | Partners | 2016P002882 |  |
| 10601 | Healthy Start | Dana Dabelea | Western IRB (WIRB) | 20181210 |  |
| 10801 | Boricua Youth Study | Cristiane Duarte | New York State Psychiatric Institute | 7377 |  |
| 10901 | Atlanta ECHO Cohort of Emory University | Anne Dunlop | Emory University | 91254 |  |
| 11001 | Safe Passage Study | Amy Elliott | WIRB  Avera IRB | 1260729; 2017.091 / 100475 |  |
| 11201 | PETALS | Assiamira Ferrara | WIRB | 20181210 |  |
| 11202 | KPRB | Assiamira Ferrara | WIRB | 20181210 |  |
| 11303 | Tucson Children's Respiratory Study | James Gern | University of Arizona IRB | 7500000005 |  |
| 11304 | Tucson Infant Immune Study | James Gern | University of Arizona IRB | 9600000328A017 |  |
| 11305 | Wisconsin Infant Study Cohort | James Gern | MCRI IRB | KEI10613 |  |
| 11306 | Childhood Origins of Asthma Study | James Gern | Health Sciences IRB | 2017-0306 |  |
| 11307 | Urban Environment and Childhood Asthma | James Gern | 1. Washington University in St. Louis IRB  2. Johns Hopkins  3. Columbia IRB  4. Boston University | 1. 201702039  2. NA_00044286/CIR00026928  NA_00025027/CIR00028419  NA_00070888/CIR00028423  3. AAAC5139  4. H-23320 |  |
| 11309 | Infant Susceptibility to Pulmonary Infections and Asthma Following RSV Exposure | James Gern | Vanderbilt University IRB | 111299 |  |
| 11310 | Epidemiology of Home Allergens and Asthma Study | James Gern | Partners | 2017P000073 |  |
| 11311 | Wayne County Health Environment Allergy and Asthma | James Gern | Henry Ford Health System | 1881 |  |
| 11312 | Childhood Allergy/Asthma Study | James Gern | Henry Ford Health System | 2855 |  |
| 11401 | MADRES | Frank Gilliland | University of Southern California | HS-15-00498 |  |
| 11601 | ReCHARGE: Revisiting CHildhood Autism Risks from Genes and the Environment Study | Irva Hertz-Picciotto | University of California Davis | 952089 |  |
| 11701 | Pittsburgh Girls Study | Alison Hipwell | University of Pittsburgh | PRO17050429 |  |
| 11801 | New Hampshire Birth Cohort Study | Margaret Karagas | Dartmouth College | CPHS20844 |  |
| 11901 | CANDLE | Catherine Karr | University of Washington | 0638 |  |
| 11902 | The Infant Development and Environment II Study | Catherine Karr | University of Washington | 0638 |  |
| 11903 | GAPPS | Catherine Karr | University of Washington | 0638 |  |
| 12101 | Early Growth and Development Study | Leslie Leve | University of Oregon | 08082016.007 |  |
| 12102 | Early Growth and Development Study - Cohort II | Leslie Leve | University of Oregon | 08082016.007 |  |
| 12103 | Early Parenting of Children | Leslie Leve | University of Oregon | 08082016.007 |  |
| 12201 | Understanding Risk Gradients from Environment on Native American Child Health Trajectories: Toxicants, Immunomodulation, Metabolic syndromes, & Metals Exposure | Johnnye Lewis | University of New Mexico Health Sciences Center  IRB Navajo Nation Human Research Review Board | (#19-423 and 11-310)  (NNR#19.360 and 11.323) |  |
| 12301 | VDAART | Augusto Litonjua | Brigham & Women’s Hospital | 2009P000557 |  |
| 12401 | Vitamin C to Decrease Effects of Smoking in Pregnancy on Infant Lung Function | Cynthia McEvoy | Oregon Health & Science University | 6091 |  |
| 12402 | In-Utero Smoke, Vitamin C, and Newborn Lung Function | Cynthia McEvoy | Oregon Health & Science University | 6091 |  |
| 12501 | Kennedy Krieger - Baby Siblings Research Consortium | Craig Newschaffer | Johns Hopkins Medicine IRB | NA_00038069 / CIR00023036 |  |
| 12502 | University of California Davis - Baby Siblings Research Consortium | Craig Newschaffer | UCDavis IRB | 243919-28 |  |
| 12503 | Autism Spectrum Disorders-Enriched Risk - University of  Washington - BRSC | Craig Newschaffer | University of Washington IRB | 00000499 |  |
| 12504 | Autism Spectrum Disorders-Enriched Risk-BRSC Universtiy of  Miami | Craig Newschaffer | University of Miami IRB | 20160954 |  |
| 12505 | University of Washington-Infant Brain Imaging Study | Stephen Dager | University of Washington IRB | 50975 |  |
| 12506 | Autism Spectrum Disorders - Enriched Risk - IBIS -  Washington University, St. Louis | Craig Newschaffer | Washington University IRB | 201611161 |  |
| 12507 | Infant Brain Imaging Study | Robert Schultz | The Children’s Hospital of Philadelphia Research Institute IRB | 07-005689 |  |
| 12508 | Infant Brain Imaging Study | Joseph Piven | University of North Carolina-Chapel Hill | 05-2293 |  |
| 12509 | Early Autism Risk Longitudinal Investigation | Heather Volk | Johns Hopkins Bloomberg School of Public Health IRB | 00002032 |  |
| 12510 | Early Autism Risk Longitudinal Investigation | Rebecca Schmidt | ECHO WIRB IRB  UCD IRB for ASD-ER  UCD IRB for EARLI | 20181210  960711 214753-32 |  |
| 12511 | Early Autism Risk Longitudinal Investigation | Lisa Croen | WIRB | 20181210 |  |
| 12512 | Autism Spectrum Disorders - Enriched Risk EARLI - Drexel  University | Craig Newschaffer | Drexel University IRB | 1609004845  1710005749 |  |
| 12513 | University of California - Markers of Autism Risk in Babies | Craig Newschaffer | ECHO WIRB IRB  UCD IRB for ASD-ER  UCD IRB for EARLI | 20181210  960711 214753-32 |  |
| 12601 | Rochester | Tom O'Connor | University of Rochester | 58456 |  |
| 12701 | Project Viva | Emily Oken | Harvard Pilgrim Healthcare | 235301 |  |
| 12901 | ARCH | Nigel Paneth | Michigan State University  Michigan Department of Health and Human Services | LEGACYC07-120  201312-03-FC |  |
| 13001 | Mothers and Newborns | Frederica Perera | Columbia University Medical Center | AAAA6110 |  |
| 13101 | Illinois Kids Development Study | Susan Schantz | University of Illinois Urbana Champaign | 09498 |  |
| 13102 | Chemicals in our Bodies | Susan Schantz | University of California, San Francisco | UCSF IRB #  13-12160 |  |
| 13201 | Utah's Children's Project | Joseph Stanford | University of Utah | 63183 |  |
| 13301 | The NYU Children’s Health and Environment Study | Leonardo Trasande | NYU Grossman School of Medicine | s15-00778, s16-01617 |  |
| 13501 | Asthma Coalition on Community, Environment & Stress | Rosalind Wright | Program for the Protection of Human Subjects at Mount Sinai | 12-00661 |  |
| 13502 | Programming of Intergenerational Stress Mechanisms | Rosalind Wright | Program for the Protection of Human Subjects at Mount Sinai | 12-00875 |  |
| 13503 | Inova Childhood Longitudinal Study | Rosalind Wright | Inova | U19-02-3423 |  |

2. We note that you indicated that there are NIH Grant numbers for each author and institution receiving grant funding that contributed to this work. However, these grant numbers are missing from your Funding Statement. Please provide all information regarding the funding of your study including grant numbers. We will update your Funding Statement with the information provided.

***Response:*** *We have provided a Supplementary File (S1 Funding) that lists the NIH grant numbers for the cohorts providing aggregate data for this study. The reference to the S1 Funding file should be included in the Funding Statement, which we have added to the Revised Manuscript (since the funding statement does not seem to be accessible as a SmartForm that we can edit).*

**ECHO Awardees and Cohorts that contributed aggregate data results for this analysis:**

| Cohort ID | Cohort Name | Cohort Contact PI | NIH Grant Number |
| --- | --- | --- | --- |
| 10101 | ECHO in Puerto Rico | Akram Alshawabkeh | UH3OD023251 |
| 10401 | 35^th^ Multicenter airway Research Collaboration | Carlos Camargo | UH3OD023253 |
| 10402 | 43^rd^ Multicenter Airway Research Collaboration | Carlos Camargo | UH3OD023253 |
| 10601 | Healthy Start | Dana Dabelea | UH3OD023248 |
| 10801 | Boricua Youth Study | Cristiane Duarte | UH3OD023328 |
| 10901 | Atlanta ECHO Cohort of Emory University | Anne Dunlop | UH3 OD023318 |
| 11001 | Safe Passage Study | Amy Elliott | UH3OD023279 |
| 11201 | PETALS | Assiamira Ferrara | UH3OD023289 |
| 11202 | KPRB | Assiamira Ferrara | UH3OD023289 |
| 11303 | Tucson Children’s Respiratory Study | James Gern | UH3OD023282 |
| 11304 | Tucson Infant Immune Study | James Gern | UH3OD023282 |
| 11305 | Wisconsin Infant Study Cohort | James Gern | UH3OD023282 |
| 11306 | Childhood Origins of Asthma Study | James Gern | UH3OD023282 |
| 11307 | Urban Environment and Childhood Asthma | James Gern | UH3OD023282 |
| 11309 | Infant Susceptibility to Pulmonary Infections and Asthma Following RSV Exposure | James Gern | UH3OD023282 |
| 11310 | Epidemiology of Home Allergens and Asthma Study | James Gern | UH3OD023282 |
| 11311 | Wayne County Health Environment Allergy and Asthma | James Gern | UH3OD023282 |
| 11312 | Childhood Allergy/Asthma Study | James Gern | UH3OD023282 |
| 11401 | MADRES | Frank Gilliland | UH3OD02328 |
| 11601 | ReCHARGE: Revisiting Childhood Autism Risks from Genes and the Environment Study | Irva Hertz-Picciotto | UH3OD023365 |
| 11701 | Pittsburgh Girls Study | Alison Hipwell | UH3OD023244 |
| 11801 | New Hampshire Birth Cohort Study | Margaret Karagas | UH3OD023275 |
| 11901 | CANDLE | Catherine Karr | UH3OD023271 |
| 11902 | The Infant Development and the Environment II Study | Catherine Karr | UH3OD023271 |
| 11903 | GAPPS | Catherine Karr | UH3OD023271 |
| 12101 | Early Growth and Development Study | Leslie Leve | UH3OD023389 |
| 12102 | Early Growth and Development Study – Cohort II | Leslie Leve | UH3OD023389 |
| 12103 | Early Parenting of Children | Leslie Leve | UH3OD023389 |
| 12201 | Understanding Risk Gradients from Environment on Native American Child Health Trajectories: Toxicants, Immunomodulation, Metabolic syndromes, & Metals Exposure | Johnnye Lewis | UH3OD023344 |
| 12301 | VDAART | AUHusto Litonjua | UH3OD023268 |
| 12401 | Vitamin C to Decrease Effects of Smoking in Pregnancy yon Infant Lunch Function | Cynthia McEvoy | UH3OD023288 |
| 12402 | In-Utero Smoke, Vitamin C, and Newborn Lunch Function | Cynthia McEvoy | UH3OD023288 |
| 12501 | Kennedy Krieger – Baby Siblings Research Consortium | Craig Newschaffer | UH3OD023342 |
| 12502 | University of California Davis - Baby Siblings Research  Consortium | Craig Newschaffer | UH3OD023342 |
| 12503 | Autism Spectrum Disorders-Enriched Risk - University of  Washington - BRSC | Craig Newschaffer | UH3OD023342 |
| 12504 | Autism Spectrum Disorders-Enriched Risk-BRSC University of  Miami | Craig Newschaffer | UH3OD023342 |
| 12505 | University of Washington-Infant Brain Imaging Study | Stephen Dager | UH3OD023342 |
| 12506 | Autism Spectrum Disorders – Enriched Risk – IBIS – University of Washington, St. Louis | Craig Newschaffer | UH3OD023342 |
| 12507 | Infant Brain Imaging Study | Robert Schultz | UH3OD023342 |
| 12508 | Infant Brain Imaging Study | Joseph Piven | UH3OD023342 |
| 12509 | Early Autism Risk Longitudinal Investigation | Heather Volk | UH3OD023342 |
| 12510 | Early Autism Risk Longitudinal Investigation | Rebecca Schmidt | UH3OD023342 |
| 12511 | Early Autism Risk Longitudinal Investigation | Lisa Croen | UH3OD023342 |
| 12512 | Autism Spectrum Disorders – Enriched Risk EARLI – Drexel University | Craig Newschaffer | UH3OD023342 |
| 12513 | University of California – Markers of Autism Risk in Babies | Craig Newschaffer | UH3OD023342 |
| 12601 | Rochester | Tom O’Connor | UH3OD023349 |
| 12701 | Project Viva | Emily Oken | UH3OD023286 |
| 12901 | ARCH | Nigel Paneth | UH3OD023285 UH3OD023328- |
| 13001 | Mothers and Newborns | Frederica Perera | UH3OD023290 |
| 13101 | Illinois Kids Development Study | Susan Schantz | UH3OD023272 |
| 13102 | Chemicals in Our Bodies | Susan Schantz | UH3OD023272 |
| 13201 | Utah’s Children’s Project | Joseph Stanford | UH3OD023249 |
| 13301 | Safe Passage Study | Leonardo Trasande | UH3OD023305 |
| 13501 | Asthma Coalition on Community, Environment & Stress | Rosalind Wright | UH3OD023337 |
| 13502 | Programming of Intergenerational Stress Mechanisms | Rosalind Wright | UH3OD023337 |
| 13503 | Inova Childhood Longitudinal Study | Rosalind Wright | UH3OD023337 |

Author Response to Journal Requirements

1. Please ensure that your manuscript meets PLOS ONE's style requirements, including those for file naming.

***Response:*** *In the revision, we have assured that the manuscript meets the style requirements and file naming requirements.*

2. Thank you for stating the following financial disclosure:

"No; the funders had no role in study design, data collection and analysis, decision to publish, or preparation of the manuscript."

At this time, please address the following queries:

a. Please clarify the sources of funding (financial or material support) for your study. List the grants or organizations that supported your study, including funding received from your institution. *In the original submission we supplied the NIH grant numbers for each author and institution receiving grant funding that contributed to this work; we have now added S1 Funding as a Supplementary file that can be referenced in our funding statement, which contains the NIH grant numbers for the participating cohorts. .*
b. State what role the funders took in the study. We reiterate that *“The funders had no role in study design, data collection and analysis, decision to publish, or preparation of the manuscript.”*
c. If any authors received a salary from any of your funders, please state which authors and which funders. *The authors received salary support from the funders named on the webform in part a.*
d. If you did not receive any funding for this study, please state: “The authors received no specific funding for this work.” N/A

3. We note that you have indicated that data from this study are available upon request. PLOS only allows data to be available upon request if there are legal or ethical restrictions on sharing data publicly. For information on unacceptable data access restrictions, please see [http://journals.plos.org/plosone/s/data-availability#loc-unacceptable-data-access-restrictions](https://nam03.safelinks.protection.outlook.com/?url=https%3A%2F%2Furldefense.com%2Fv3%2F__http%3A%2Fjournals.plos.org%2Fplosone%2Fs%2Fdata-availability*loc-unacceptable-data-access-restrictions__%3BIw!!OToaGQ!_qWRM0ikb2ELtvFfmku-RIKAizGrgasGsQZnHgv2cS7BCT0Abw7mdFyspV4WTqEt1v5Uiw%24&data=02%7C01%7Camlang%40emory.edu%7C3d23acbaaa7b4f1c5c7508d82e7a2177%7Ce004fb9cb0a4424fbcd0322606d5df38%7C0%7C0%7C637310450678138451&sdata=9AiRI9aeSd0cIJrc%2BsutUsDK3DTV%2FvljYcoQgEOiXVI%3D&reserved=0).

In your revised cover letter, please address the following prompts:

a) If there are ethical or legal restrictions on sharing a de-identified data set, please explain them in detail (e.g., data contain potentially identifying or sensitive patient information) and who has imposed them (e.g., an ethics committee). Please also provide contact information for a data access committee, ethics committee, or other institutional body to which data requests may be sent.

***Response:*** *As described in the manuscript, this research consists of a disseminated meta-analysis and, as such, the data analysts did not use any participant-level data (de-identified or identifiable) and do not have a participant-level data set (which was the rationale for doing the disseminated meta-analysis). The data analysts performing the disseminated meta-analysis used a cohort-level data set that contains the point estimates and variance-covariance matrices from each participating cohort. We could make this cohort-level data set available if this were of interest. As stated in the original submission, the de-identified, cohort-level data will be made available upon request (through the contact information stated in the original submission) for the following reason: This disseminated meta-analysis of extant data available from cohorts participating in the ECHO consortium reflects a range of cohorts with varying consent forms and institutional review board requirements/allowances for sharing data. Also, the Steering Committee of the ECHO consortium established guidelines that any use of the ECHO cohort data by investigators outside of ECHO would require an application, review, and approval process.*

b) If there are no restrictions, please upload the minimal anonymized data set necessary to replicate your study findings as either Supporting Information files or to a stable, public repository and provide us with the relevant URLs, DOIs, or accession numbers. Please see [http://www.bmj.com/content/340/bmj.c181.long](https://nam03.safelinks.protection.outlook.com/?url=https%3A%2F%2Furldefense.com%2Fv3%2F__http%3A%2Fwww.bmj.com%2Fcontent%2F340%2Fbmj.c181.long__%3B!!OToaGQ!_qWRM0ikb2ELtvFfmku-RIKAizGrgasGsQZnHgv2cS7BCT0Abw7mdFyspV4WTqH1Ly9W8g%24&data=02%7C01%7Camlang%40emory.edu%7C3d23acbaaa7b4f1c5c7508d82e7a2177%7Ce004fb9cb0a4424fbcd0322606d5df38%7C0%7C0%7C637310450678148446&sdata=Bgtl0INwJul7FVhnsQXsV5b7oJ8o%2BjJ2F0h3XfOgdEU%3D&reserved=0) for guidelines on how to de-identify and prepare clinical data for publication. For a list of acceptable repositories, please see [http://journals.plos.org/plosone/s/data-availability#loc-recommended-repositories](https://nam03.safelinks.protection.outlook.com/?url=https%3A%2F%2Furldefense.com%2Fv3%2F__http%3A%2Fjournals.plos.org%2Fplosone%2Fs%2Fdata-availability*loc-recommended-repositories__%3BIw!!OToaGQ!_qWRM0ikb2ELtvFfmku-RIKAizGrgasGsQZnHgv2cS7BCT0Abw7mdFyspV4WTqGlvLOPQg%24&data=02%7C01%7Camlang%40emory.edu%7C3d23acbaaa7b4f1c5c7508d82e7a2177%7Ce004fb9cb0a4424fbcd0322606d5df38%7C0%7C0%7C637310450678148446&sdata=TUqgrtn4wa5Fupd%2FFuBAemOxYZozl%2B4GdzSeQBd%2BA40%3D&reserved=0). *N/A*

4. One of the noted authors is a group or consortium [Environmental Influences on Child Health Outcomes]. In addition to naming the author group, please list the individual authors and affiliations within this group in the acknowledgments section of your manuscript. Please also indicate clearly a lead author for this group along with a contact email address.

***Response:*** *The individual authors that are part of the consortium are listed in the Acknowledgements. We have now named two lead authors for this group and provided their e-mail addresses.*

5. We note that you have included the phrase “data not shown” in your manuscript. Unfortunately, this does not meet our data sharing requirements. PLOS does not permit references to inaccessible data. We require that authors provide all relevant data within the paper, Supporting Information files, or in an acceptable, public repository. Please add a citation to support this phrase or upload the data that corresponds with these findings to a stable repository (such as Figshare or Dryad) and provide and URLs, DOIs, or accession numbers that may be used to access these data. Or, if the data are not a core part of the research being presented in your study, we ask that you remove the phrase that refers to these data.

***Response:*** *As these data are not a core part of the research being presented, we removed the phrase that refers to these data in the Results Section of the Revised Manuscript (page 23).*

6. We note that Figure 1 in your submission contain [map/satellite] images which may be copyrighted. All PLOS content is published under the Creative Commons Attribution License (CC BY 4.0), which means that the manuscript, images, and Supporting Information files will be freely available online, and any third party is permitted to access, download, copy, distribute, and use these materials in any way, even commercially, with proper attribution. For these reasons, we cannot publish previously copyrighted maps or satellite images created using proprietary data, such as Google software (Google Maps, Street View, and Earth). For more information, see our copyright guidelines: [http://journals.plos.org/plosone/s/licenses-and-copyright](https://nam03.safelinks.protection.outlook.com/?url=https%3A%2F%2Furldefense.com%2Fv3%2F__http%3A%2Fjournals.plos.org%2Fplosone%2Fs%2Flicenses-and-copyright__%3B!!OToaGQ!_qWRM0ikb2ELtvFfmku-RIKAizGrgasGsQZnHgv2cS7BCT0Abw7mdFyspV4WTqEbe7NDwg%24&data=02%7C01%7Camlang%40emory.edu%7C3d23acbaaa7b4f1c5c7508d82e7a2177%7Ce004fb9cb0a4424fbcd0322606d5df38%7C0%7C0%7C637310450678158438&sdata=4Yf6mXGJYlTKF7yS7saFKlrj%2BQbklYThkoErwtz7cTI%3D&reserved=0).

We require you to either (1) present written permission from the copyright holder to publish these figures specifically under the CC BY 4.0 license, or (2) remove the figures from your submission:

6.1.    You may seek permission from the original copyright holder of Figure 1 to publish the content specifically under the CC BY 4.0 license.

***Response:*** *The ECHO Data Analysis Center (DAC) created Figure 1 specifically for this manuscript, and there is no copyright on it. Figure 1 has a unique identifier on the map (ECHO2020-1002) to differentiate other ECHO-generated maps from this non-copyrighted map.*

**Reviewer #1 Comments to the Author**

1. Could the authors provide more information on ECHO cohorts and how a specific cohort could be included? Given the epidemiology nature of these analyses, the focus on broad geographic regions, and that more than 20 cohorts are represented, I was surprised that the sample size was relatively small (~ 25,000). For example, I am aware of epidemiology cohorts that are representative of wide geographic regions and that would meet the author’s inclusion criteria and that have at least 1,000,000 or more participants each. Given that sample size was cited as a potential limitation and reason for why some effects were not detected, it would be valuable for the authors to provide more context for the ECHO project, and the novel use of ECHO project data relative to this question specifically.

***Response:*** *In the Objective section of the Revised Manuscript (page 7), we have added more information to describe the ECHO consortium of cohorts and how they were selected (and reference an article that goes into more detail on this topic). We also added language to the Objective section (pages 7 & 8) to introduce that ECHO uses both extant data (in collective analyses, such as this) and a common protocol to collect new data.*

2. The authors focused on maternal education as an indicator of individual-level SES. Could the authors comment on whether they would expect different or similar patterns of findings if other SES indicators were available? For example, one strategy is to get mother and partner SES indicators, and take the highest as the indicator (even if mothers have low education, the status of their more-educated partner could increase their status by proxy), but only mother education level is available here. Other studies focus on income and occupation status as individual-level indicators. Given that the authors discuss how the benefits of education can vary by race/ethnicity, e.g. lower salary despite equivalent education attainment for Black individuals, it would be valuable to comment on this.

***Response:*** *In the Introduction section of the Revised Manuscript (page 6) we provided a statement indicating that the literature supports that across “various measures of individual-level SES (including maternal level of education and income, marital and employment status, and type of health insurance) there is a consistent social gradient in the risk of preterm birth.”*

*In the Methods section of the Revised Manuscript (page 10), we also added the following language: “We used the categorical variable of maternal level of education as the main individual-level measure of SES based on existing research among US populations that support that education is the dimension of SES that most strongly and consistently predicts health, especially for women and children (references 35, 36). Also, maternal level of education as a categorical variable was more available across the ECHO cohort extant data. Paternal level of education was less often collected by the cohorts or, when collected, was oftentimes missing. Similarly, household income was less available across cohorts in that either entire cohorts did not collect this data or the variable response was missing within cohorts. Also, in order to be meaningfully interpreted income in relation to the federal poverty line, household income must be combined with household density, which was also not uniformly available across cohorts and/or was missing within cohort extant data. Furthermore, even when using the federal poverty thresholds, the meaning of the various income groupings varies a great deal across states and according to rural/urban status.”*

3. One criticism that I have seen of using a categorical approach to education is that racial/ethnic distributions within each category might not be equivalent. For example, when grouping bachelors or higher individuals, even within that category White individuals could be higher SES (e.g. more likely to have post-graduate training) compared to Black individuals (more likely to have a bachelor’s degree). Using continuous variables could reduce the risk of this potential confound. Why did the authors chose to use categorical rather than continuous variables? And why were these specific categories chosen?

***Response:*** *See our Response to Reviewer #1, Comment #2, in which we address this issue (and page 10 of the Revised Manuscript). To summarize, the extant data on level of education available from the participating cohorts was collected data as a categorical variable, thus, categorical data is what was available across cohorts. The categorization of maternal education data reflected how cohorts collected and categorized the data, and is based on accepted categories of attainment (including those references in the manuscript that pertain to research within US populations).*

4. The census-level tract analyses were interesting. Could the authors provide more justification or context for their use of those particular indicators? For example, I thought that percent Black population per census tract was an interesting choice, especially given that non-Black, non-White races/ethnicities were also considered. Is percent Black individuals a particularly valuable indicator of neighbourhood-level SES for other non-White races/ethnicities, or would percent of other races/ethnicities also potentially matter?

***Response:*** *In the Methods section of the Revised Manuscript (page 11), we have inserted parenthetical phrases indicating that we included the percentage black population in the census tract as a measure of segregation and the percentage population living below the poverty line as a measure of poverty. Also, in the Methods section of the Revised Manuscript (page 12) we have explained and referenced our rationale for selecting the single indicator measure of neighborhood-level SES rather than more complex indices. Specifically, we modeled our selection of census tract variables on the paper by Carmichael et al. (2017), who investigated the association between percent of the tract population with household income below the poverty level (as measure of neighborhood-level deprivation) and percent of the tract population that was black (as a measure of segregation) and preterm birth; in their analysis, the Carmichael paper also created an index that incorporated eight census tract variables representing multiple aspects of socioeconomic level following methods of Messer et al. (2006). The Carmichael paper found that the single measure of tract poverty correlated well with the multi-measure index (r = 0.86) and resulted in the same conclusions. Therefore, we chose to focus on the single indicator measures, rather than on the more complex indices, as in Carmichael et al. (2017), Krieger et al. (2003 and 2005), and Rehkopf et al. (2006).*

5. The authors used a stratification approach to look at differences in the association between education and gestational length by race/ethnicity, geographic region, etc. Did the authors test any interaction terms or conduct any omnibus tests to determine if, overall, these differences in groups or moderating effects were statistically significant?

***Response:*** *We did not test the significance of interaction terms nor conduct omnibus tests to determine if, overall, the observed differences were statistically significant. An interaction between maternal education, gestational age and region was not possible as most participating cohorts contributed to one geographic region only. No individual-level data was available to the ECHO Data Analysis Center (DAC) so all analyses needed to be conducted successfully at each specific cohort, and then meta-analyzed at the DAC. In the Revised Manuscript, in the Objectives and Methods sections, we have emphasized that this analysis reflects a disseminated meta-analysis of extant data from the ECHO cohorts performed before individual-level data could be shared across cohorts.*

6. Given that differences did emerge for the Non-Hispanic Other Race category, could the authors provide a detailed racial/ethnic breakdown of this group in the supplemental materials? Although the focus has traditionally been on Black and Hispanic racial/ethnic groups, this information could be helpful for researchers examining health disparities in other racial/ethnic groups.

***Response:*** *In the Revised Manuscript we reference and include a Supplemental Table (S2; page 21), which is a detailed racial distribution by gestational age category, to include the following race categories: White, Black, Asian, Native Hawaiian or Other Pacific Islander, American Indian or Alaska Native, Multiple race, other race. We did not calculate race/ethnicity-specific effect estimates for these additional categories as our cohort-specific sample sizes were too small resulting in unstable estimates. We have also included information providing the ethnicity distribution by gestational age category.*

7. There were some interesting findings by geographic region and rurality of residence, but my first thought was how much overlap is there in these constructs? Could neighbourhood SES indicators be conflated by geographic region? It would be helpful if the authors included breakdowns of race/ethnicity and neighbourhood-level SES indicators by geographic region, at least in supplemental materials. Or if the authors included other SES indicators as covariates in regional analyses, that should be clearly stated in conclusions.

***Response:*** *In the Results section of the Revised Manuscript (page 25), we have included reference to a Supplemental Table (S4), which displays the distribution of race/ethnicity and neighborhood-level SES by geographic region. We did not include other SES indicators as covariates in regional analyses. To the Discussion section of the Revised Manuscript (page 26), we also noted that it is possible that neighborhood SES is conflated by geographic region. In the Discussion section, we also note that future ECHO analyses utilizing individual-level participant data will allow these complex relationships to be explored.*

8. Could the authors provide more comment on the regional differences detected in the discussion? For example, are there regional differences in terms of laws or regulations that affect access to health care or education? Are there differences in societal or governmental wealth between these regions, or urban verses rural? And could these factors be relevant? The authors note that college education is rarer in the Midwest and South – are they suggesting that, as a consequence, individuals who do attain a college education in these regions are comparatively more privileged and affluent than similarly educated people in the West or Northeast?

***Response:*** *To the Discussion section of the Revised Manuscript (page 26), we have added a brief discussion and citations to full reports that discuss how the variation in state-level policies that relate to maternal and child health and health outcomes.*

**Reviewer #2 Comments to the Author**

1. Introduction: There are many existing studies focusing on SES and preterm delivery. More details are needed to explain what added values this study brings to the field and what limitations from previous studies this study can address.

***Response:*** *In the Objective section of the Revised Manuscript (pages 7 & 8), we have added narrative to further emphasize the importance of combining extant data across ECHO cohorts to examine associations by varying US region as well as the innovativeness of using the collective analyses (disseminated meta-analysis) process to combine extant data across cohorts when individual-level data cannot be shared. The Discussion section (Strengths and Limitations part) includes a discussion of the strengths and contributions of this literature to the existing literature, which may be better interpreted now that the Revised Manuscript better describes the ECHO cohorts and the use of extant data.*

2. Geocoding: is residential history during pregnancy available? Why only using the earliest address collected during pregnancy?

***Response:*** *Most of the cohorts participating in this disseminated meta-analysis of extant data did not collect residential history during pregnancy, but only collected the earliest address during pregnancy. We chose to use the earliest address during pregnancy because it was more available across participating cohorts.*

3. Neighborhood-level SES variables: census tract is not ideal to assess SES as numerous findings have shown that there can be large heterogenities in SES within a census tract. Please use census block group instead.

***Response:*** *In the Methods section of the Revised Manuscript (page 12), we provide an explanation and citations for our use of census tract level measures of SES. Briefly,* we chose to base our neighborhood-level SES measures on census tract based on the existing literature that shows that among non-Hispanic Black, non-Hispanic White, and Hispanic men and women, measures of economic deprivation were most sensitive to expected socioeconomic gradients in health, with the most consistent results and maximal geocoding linkage evident for tract-level analyses. Furthermore, census tract–level analyses yielded the most consistent results with maximal geocoding linkage (i.e., the highest proportion of records both geocoded and linked to census-defined areas) (Krieger et al., 2003; Krieger et al., 2005).

Furthermore, we chose to use DeGAUSS system for geocoding for the following reasons: *it is a freely available distributed geocoding system that used the same nationwide underlying street address database (the Census Bureau’s Topologically Integrated Graphical Encoding and Referencing “TIGER” data); a single geocoding engine (embedded in DeGAUSS) resulting in all cohorts using the same underlying address matching code; and a single, uniform underlying street database (TIGER). A centralized DAC geocoding process was not possible since the DAC did not have access to individual-level data. Purchasing, installing, and executing a commercially-available geocoder at all cohort locations was not possible.*

4. Line 225: why only 2005-2009 ACS was used? The selection of specific ACS data should correspond to women's pregnancy period: e.g., you can use the middle year of the 5-year ACS as the index year, and generate time-weighted averages for each woman.

***Response:*** *To the Methods section of the Revised Manuscript (page 12) we also added narrative to describe why we chose to use the five-year ACS data for the period 2005-2009.*

5. Lines 225-226: it is unclear why only three variables were used. Several well-established SES indices such as the Neighborhood Deprivation Index or Area Deprivation Index can be used to characterize SES, which are usually derived based on dozens of variables.

***Response:*** *See our response to Reviewer #1, Comment #4.*

6. Covariates: why income was not included? In addition, is there any information on gestation week when prenatal care started? The binary prenatal care status won't tell much as the majority of women received prenatal care.

***Response:*** *See our response to Reviewer #1, Comment #2. Also, across the extant data available within the cohorts, the gestational week at which prenatal care was initiated was not widely available and/or was not collected in a similar manner (by self-report vs. by medical record abstraction).*

7. Statistical modeling: it is unclear why the authors run separate analyses on each individual cohort and then pool the results together using a meta-analysis. This is usually done when people don't have direct access to the raw data from each site. The authors may confirm whether this is the case. Otherwise, the authors should use a mixed effects model including data from all sites with random intercepts by cohort and by census block group.

***Response:*** *In the Objective section of the Revised Manuscript (pages 7 and 8), we have added more information to describe the ECHO consortium’s plan to utilize collective analyses (disseminated meta-analysis) to combine data across cohorts for hypothesis-testing when identifiable participant-level data could not be shared. The authors did not have access to individual-level data, therefore each cohort performed cohort-specific analyses based on specifications provided to them by the DAC, and the DAC pooled the results together using a meta-analysis.*

8. In addition to the multinomial logistic regression, the authors may also treat the gestational age as a continuous outcome and model it use linear regression.

***Response:*** *In the Discussion section of the Revised Manuscript, we did add a statement specifying that in the future ECHO studies will involve individual-level data analyses examining gestational age as a continuous variable (page 30). Because this study represents a disseminated meta-analysis in which no participant-level data was held by the Data Analysis Center (DAC), the DAC only received the point estimates and variance/co-variance matrix from each cohort’s own local analysis. The a priori analysis specified the use of categorical gestational age to estimate the associations.*

9. Limitation: Another limitation is the potential selection bias. It is not clear how representative the study samples are. There is no site in TX and FL, the states with the second and third most population in the US.

***Response:*** *See our response to Reviewer #1, Comment #1.*
