# Supplementary material for: Racial and geographic variation in effects of maternal education and neighborhood-level measures of socioeconomic status on gestational age at birth: Findings from the ECHO cohorts
Source: PLoS One. 2021 Jan 8;16(1):e0245064. doi: 10.1371/journal.pone.0245064 (PMC7794036; doi:10.1371/journal.pone.0245064)
Supplement: Attachment — Submitted filename: Response to Review R2_submitted.docx [file pone.0245064.s008.docx]

PONE-D-20-14238R2
Racial and Geographic Variation in Effects of Maternal Education and Neighborhood-level Measures of Socioeconomic Status on Gestational Age at Birth: Findings from The ECHO Cohorts
PLOS ONE - **Author Response to Review**

Author Response to Reviewer #2

1. Reviewer 2: The current analyses did not fully leverage the potential of the ECHO study. The same analyses can be easily done using birth certificate data with larger sample sizes and minimal selection bias. It is unclear what the added value this study brings to the field. The idea of collective analysis is not new. It has been widely used in other settings: e.g., pooled time-series analyses on air pollution studies.

Author Response: We respectfully disagree with this Reviewer comment for the following reasons:

(1) The same analysis cannot easily be done with birth certificate data because in using publicly-available birth certificate data (available for all states via the National Center for Health Statistics) it is not possible to obtain a unit of geography smaller than county or zip code of maternal residence (and these are only for units with population > 100,000). *Specifically, maternal census tract of residence (the geographic level of interest for this manuscript) is not publicly-available through National Center for Health Statistics, prohibiting the conduct of census-tract level analyses on a multi-stage large-scale basis with publicly available data, which we were able to accomplish with the ECHO data*. While for a given state it would be possible to make a request to the state’s department of public health to obtain and enter into a data use agreement to use census tract-level geocodes for that state, this would be very time-consuming to accomplish across multiple states and, furthermore, many states simply do not share geocodes at the census tract-level. Thus, we believe that this analysis using census tract-level data available within the ECHO cohort makes a substantial contribution to the literature.

(2) Within the published literature, there is not another disseminated meta-analysis that has brought together extant data from existing pediatric cohorts to address the research question of how individual- *and* neighborhood-level markers of socioeconomic status associate with gestational age at birth across the United States. Existing literature that has considered individual- and neighborhood-level markers of socioeconomic status have mostly focused in a *single state* or smaller geography of the United States, rather than including multiple regions across the United States, as this multi-cohort research does.

In summary, we believe that this work does represent a unique manner of collaboration in which the existing pediatric cohorts came together through ECHO to perform a disseminated meta-analysis upon their extant data under the guidance of a central Data Analysis Center. *Thus, both the findings of this research, around the role of individual- and neighborhood-level markers of socioeconomic status on gestational age at birth, as well as the methods of the use of data from multiple cohorts that do not yet have permission to share data, are of interest to readership.*

However, to address this important concern of the Reviewer, we have added a statement to the Discussion section of the R2 Manuscript (pages 28-29): *“Another strength of the ECHO-wide cohort data is the availability of maternal address at the cohort level, which allowed for geocoding and assignment of neighborhood-level measures of SES at the CT level. Publicly-available birth record data does not allow for discernment of geography to the level of CT. Within the published literature, there is not another study that has brought together extant data from multiple pediatric cohorts or across multiple states to address whether individual- and neighborhood-level markers of socioeconomic status associate with gestational age at birth across the United States. Existing literature that has considered individual- and neighborhood-level markers of socioeconomic status have mostly focused within a single state or has been carried out at a geographic level larger than CT (such as city or county).”*

2. Reviewer 2: Regarding the neighborhood SES variables: the authors stated that "census tract–level analyses yielded the most consistent results with maximal geocoding linkage (i.e., the highest proportion of records both geocoded and linked to census defined areas)". If the geocoding was performed using residential address, I don't understand why you can find the corresponding census tract but not the census block group.

Author Response: In our previous Response to Review and R1 manuscript, we included this statement: *“We chose to consider SES measures at the level of census tract based on literature showing that among non-Hispanic black, non-Hispanic white and Hispanic men and women, measures of economic deprivation were most sensitive to socioeconomic gradients in health with the most consistent results and maximal geocoding evidence for census tract-level analyses (Krieger et al., 2003; Krieger et al., 2005).”* This statement, about the maximal geocoding linkage, was referring to the experience of the authors of those manuscripts (not to our experience in the ECHO data). Also, this referenced statement was intended to provide rationale for why we chose to focus our analysis at the level of census tract (i.e., that literature supports that measures of economic deprivation at that level were most consistently associated with gradients in health outcomes).

To clarify any confusion and address this Reviewer concern further, we have edited this statement in the Methods section of the R2 Manuscript (pages 12-13) to more clearly convey those ideas: *“We chose to consider SES measures at the level of CT based on literature showing that CT-level analyses resulted in maximal geocoding linkage (i.e., the highest proportion of records geocoded and linked to census-defined geography) and that measures of economic deprivation at the CT-level were most sensitive to expected socioeconomic gradients in health among non-Hispanic black, non-Hispanic white and Hispanic men and women (Krieger et al., 2003; Krieger et al., 2005).*

3. The authors did not answer my question why only 3 variables were selected, and why other well-established SES indices were not used.

Author Response: In our *previous* Response to Review, we had replied to this question with the following narrative addition to the R1 Manuscript (page 12),: *“We selected the three CT level markers of SES based on past research that has investigated the relationship between census tract markers of SES and preterm birth, which found that the single-measure variable of percentage of persons below poverty performed as well as more complex, composite measures of economic deprivation [23]  in conjunction with our a priori interest in exploring census tract markers of rural/urban status and segregation in conjunction with poverty.”*

In the R2 Manuscript (page 12) we have added further detail to that previous response (the underlined stanza) to more thoroughly clarify our rationale for not using an established index: *“We selected the three CT level markers of SES based on past research that investigated the relationship between census tract markers of SES and preterm birth, which found that the single-measure variable of percentage of persons below the poverty level performed as well as more complex, composite measures of economic deprivation [23] in conjunction with our a priori interest in exploring CT markers of rural/urban status and segregation in conjunction with poverty. Specifically, Carmichael et al. [23] found that the single-measure of percent of the CT population below the poverty level correlated well with an eight-measure index of CT variables (r=0.86) and resulted in the same conclusions.”*

4. The authors did not address my question why only the 2005-2009 ACS was used. ACS starts to provide 5-year estimate data since 2005-2009, and the latest data available is 2014-2018. There can be large temporal changes in the SES indicators in certain areas across the years, which cannot be addressed if only one 5-year estimate data is used.

Author Response: In our *previous* Response to Review we did reply to this question by including a narrative response in the R1 manuscript (page 13): *“We used five-year data releases from ACS as only the five-year releases are guaranteed to have estimates for every CT in the United States. In this disseminated meta-analysis, addresses were geocoded at the cohort level using DeGAUSS since participant address could not be shared with the DAC; disseminating analytical code to the cohorts to run locally using time-weighted averages for each woman was not possible across all of the participating cohorts.”*

We acknowledge, however, that this response did not completely capture the concern of the Reviewer. As such, in the R2 Manuscript, we have done the following:

a. To the Methods Section of the R2 Manuscript (pages 10-11), we have added further detail to our rationale for using the DeGAUSS tool for the disseminated geocoding across multiple cohorts and specifically detailed why we choose the 2005-2005 ACS 5-year release (pages 10-11), which now reads as follows: *“DeGAUSS, a freely available distributed geocoding system, facilitates reproducible geocoding and geomarker assessment in multi-site studies while maintaining the confidentiality of protected health information by enabling the sites access to a single embedded geocoding engine that uses the same nationwide street address data base (the Census Bureau’s Topologically Integrated Graphical Encoding and Referencing, TIGER, data) and matching code without having to share address data. The DeGAUSS geocoder used current (at the time of the study) TIGER street centerline address range files to convert residential addresses into geographical coordinates and provided assessment of qualitative precision for the geocodes [(37)]. Using the latest TIGER street centerline address range files was appropriate because of improvements to street and address ranges made by census bureau over time. After geocoding, the resulting latitude/longitude coordinates were joined to the TIGER 2000 Census Tract boundaries. Since census tracts are revised only every ten years, the TIGER 2000 Census Tract boundaries were needed in order to correctly match the census tract for geocoded addresses to the 2005-2009 American Community Survey (ACS) 5-year estimates, which used the same census tract identifiers as the 2000 TIGER census tract layer. We chose the ACS 2005-2009 dataset because for births prior to 2005-2009 there is not a corresponding five-year ACS file that contains estimates for every CT (2005-2009 is the first available release of this data) and, during the analysis planning stage, 2008 seemed to be the approximate mid-point of participant enrollment.”*
b. To the Discussion/Limitations Section of the R2 Manuscript (page 29) we have added text acknowledging that a limitation of our approach is the use of a single 5-year estimate across all births for a given cohort: *“A limitation related to the assignment of CT level variables via ACS is that we used a single 5-year file across all cohorts (the 2005-2009 five-year release) including for birth years before or after that period, which could have resulted in some misclassification given that there could have been temporal changes in the SES indicators in some areas that would not have been captured by using the 2005-2009 year period. For births before 2005-2009, there is not a corresponding five-year ACS file that contains estimates for every CT (2005-2009 is the first available release of this data). While there is a five-year release for data after that period, we chose the ACS 2005-2009 dataset because, during the analysis planning stage, 2008 seemed to be the approximate mid-point of participant enrollment. Furthermore, it was deemed infeasible for us to use multiple five-year data releases as this would have required complex revisions and additions to the DeGAUSS tool, dissemination of analytic code to the cohorts to run locally using time-weighted averages, as well as extensive training of local cohort personnel.”*

We believe that the strengths of the study far outweigh this limitation and the work represents a meaningful contribution to the literature in that within the published literature, there is not another disseminated meta-analysis that has brought together extant data from existing pediatric cohorts to address the research question of how individual- and neighborhood-level markers of socioeconomic status associate with gestational age at birth across the United States. Existing literature that has considered individual- and neighborhood-level markers of socioeconomic status have mostly focused in a single state or smaller geography of the United States, rather than including multiple regions across the United States, as this research does.

Thank you for this opportunity to respond to the review.
